# Supplementary material for: A platform incorporating trimeric antigens into self-assembling nanoparticles reveals SARS-CoV-2-spike nanoparticles to elicit substantially higher neutralizing responses than spike alone
Source: Sci Rep. 2020 Oct 23;10:18149. doi: 10.1038/s41598-020-74949-2 (PMC7584627; doi:10.1038/s41598-020-74949-2)
Supplement: Supplementary file 1 — Supplementary Table S1. [file 41598_2020_74949_MOESM1_ESM.pdf]

A Platform Incorporating Trimeric Antigens into Self-Assembling Nanoparticles Reveals SARS-CoV-2-Spike Nanoparticles to Elicit Substantially Higher Neutralizing Responses than Spike Alone

Baoshan Zhang[1*], Cara W. Chao[1*], Yaroslav Tsybovsky[2], Olubukola M. Abiona[1], Geoffrey B. Hutchinson[1], Juan I. Moliva[1], Adam S. Olia[1], Amarendra Pegu[1], Emily Phung[1,3], Guillaume B. E. Stewart-Jones[1], Raffaello Verardi[1], Lingshu Wang[1], Shuishu Wang[1], Anne Werner[1], Eun Sung Yang[1], Christina Yap[1], Tongqing Zhou[1], John R. Mascola[1], Nancy J. Sullivan[1], Barney S. Graham[1], Kizzmekia S. Corbett[1] and Peter D. Kwong[1¶]

[1] Vaccine Research Center, National Institute of Allergy and Infectious Diseases, National Institutes of Health, Bethesda, Maryland, USA.

[2] Electron Microscopy Laboratory, Cancer Research Technology Program, Leidos Biomedical Research Inc., Frederick National Laboratory for Cancer Research, Frederick, Maryland, USA.

[3] Institute for Biomedical Sciences, George Washington University, Washington, DC, USA.

[*] These authors contributed equally to this work.

¶ Corresponding author (PDK) Email: pdkwong@nih.gov

Supplementary Table S1. Amino acid sequences of constructs for protein expression.

Construct name	Amino acid sequence
LuS-N71-SpyTag	MDSKGSSQKGSRLLLLLVVSNLLLPQGVVGAHIVMVDAYKPTKGSGSAMQIYEGKLTAEGLRFGIVASRFNHALVDRL<br>VEGAIDAIVRHGGREEDITLVRVPGSWEIPVAAGELARKENISAVIAIGVLIRGATPHFDYIASEVSKGLADLSLELRKPIT<br>FGVITADTLEQAIERAGTKHGNKGWEAALSAIEMANLFKSLRGGLVPRGSHHHHHHSAWSHPQFEK
Ferritin-N96-SpyTag	MDSKGSSQKGSRLLLLLVVSNLLLPQGVVGQHHHHHHHHSAWSHPQFEKGGLVPRGGAHIVMVDAYKPTKGGGSG<br>DPMLSKDIIKLLNEQVNKEMQSSNLYMSMSSWCYTHSLDGAGLFLFDHAAEEYEHAKKLIIFLNENNVPVQLTSISAPE<br>HKFEGLTQIFQKAYEHEQNISESINNIVDHAIKSKDHATFNFLQWYVAEQHEEEVLFKDILDKIELIGNENHGLYLADQY<br>VKGIAKSRKS
RSV F-SpyCatcher	MELLILKANAITTILTAVTFCFASGQNITEEFYQSTCSAVSKGYLGALRTGWYTSVITIELSNIKEIKCNGTDAKVKLIKQE<br>LDKYKNAVTDLQLLMQSTPATGSGSAIASGVAVCKVLHLEGEVNKIKSALLSTNKAVVSLSGCGVSVLTFKVLDLKNYI<br>DKQLLPILNKQSCSIPNIETVIEFQQKNNRLLEITREFSVNAGVTTPVSTYMLTNSELLSLINDMPITNDQKKLMSNNVQI<br>VRQQSYSIMCIIKEEVLAYVVQLPLYGVIDTPCWKLHTSPLCTTNTKEGSNICLTRTDRGWYCDNAGSVSFFPQAETC<br>KVQSNRVFCDTMNSRTLPSEVNLCNVDIFNPKYDCKIMTSKTDVSSSVITSLGAIVSCYGKTKCTASNKCRGIIKTFSN<br>GCDYVSNKGVDTVSVGNTLYYVNKQEGQSLYVKGEPIINFYDPLVFPSDEFDASISQVNEKINQSLAFIRKSDELLSAIG<br>GYIPEAPRDGQAYVRKDGEWVLLSTFLGSGDSATHIKFSKRDEDGKELAGATMELRDSSGKTISTWISDGQVKDFYL<br>YPGKYTFVETAAPDGYEVATAITFTVNEQGQVTVNGKATKGDAHIGSGLVPRGSHHHHHHSAWSHPQFEK
PIV3 F-SpyCatcher	MYSMQLASCVTLTLVLLVNSQIDITKLQHVGVLVNSPKGMKISQNFETRYLILSLIPKIEDSNSCGDQQIKQYKRLLDRLII<br>PLYDGLKLQKDVIVTNQESNENTDPRTERFFGGVIGTIALGVATSAQITAAVALVEAKQAKSDIEKLKEAIRDTNKAVQS<br>VCSSVGNCIVAIKSVQDYVNKEIVPSIARLGCEAAGLQLGIALTQHYSELTNCFGDNIGSLQEKGIKLQCIASLYRTNITEI<br>FTTSTVDKYDIYDLLFTESIKVRVIDVDLNDYSITLQVRLPLLTRLLNTQIYKVDSISYNIQNREWYIPLPSHIMTKGAFLG<br>GADVKECIEAFSSYICPSDPGFVLNHEMESCLSGNISQCPRTTVTSDIVPRYAFVNGGVVANCITTTCTCNGIGNRINQ<br>PPDQGVKIITHKECNTIGINGMLFNTNKEGTLAFYTPDDITLNNSVALDPIDISIELNKVKSDLEESKEWYRRSNQKLSAI<br>EDKIEEILSKIYHIENEIARIKKLIGEAPGGSGGDSATHIKFSKRDEDGKELAGATMELRDSSGKTISTWISDGQVKDFYL<br>YPGKYTFVETAAPDGYEVATAITFTVNEQGQVTVNGKATKGDAHIGSGLVPRGSHHHHHHSAWSHPQFEK
SARS-CoV-2 spike-SpyCatcher*	MGWSCIILFLVATATGVHSAPELLGGPSVFLFPPKPKDTLMISRTPEVTCVVVDVSHEDPEVKFNWYVDGVEVHNAKT<br>KPREEQYNSTYRVVSVLTVLHQDWLNGKEYKCKVSNKALPAPIEKTISKAKGQPREPQVYTLPPSRDELTKNQVSLY<br>CLVKGFYPSDIAVEWESNGQPENNYKTTPPVLDSDGSFFLYSKLTVDKSRWQQGNVFSCSVMHEALHNHYTQKSLS<br>LSPGKGGGGSGGGGSGGGGSGGGGSAPELLGGPSVFLFPPKPKDTLMISRTPEVTCVVVDVSHEDPEVKFNWYVD<br>GVEVHNAKTKPREEQYNSTYRVVSVLTVLHQDWLNGKEYKCKVSNKALPAPIEKTISKAKGQPREPQVYTLPPSRDE<br>LTKNQVSLTCLVKGFYPSDIAVEWESNGQPENNYKTTPPVLDSDGSFFLTSKLTVDKSRWQQGNVFSCSVMHEALH<br>NHYTQKSLSLSPGKGGSGGGGSGGLEVLFQGPQCVNLTTRTQLPPAYTNSFTRGVYYPDKVFRSSVLHSTQDLFLP<br>FFSNVTWFHAIHVSGTNGTKRFDNPVLPFNDGVYFASTEKSNIIRGWIFGTTLDSKTQSLLIVNNATNVVIKVCEFQFC<br>NDPFLGVYYHKNNKSWMESEFRVYSSANNCTFEYVSQPFLMDLEGKQGNFKNLREFVFKNIDGYFKIYSKHTPINLV<br>RDLPQGFSALEPLVDLPIGINITRFQTLLALHRSYLTPGDSSSGWTAGAAAYYVGYLQPRTFLLKYNENGTITDAVDCA<br>LDPLSETKCTLKSFTVEKGIYQTSNFRVQPTESIVRFPNITNLCPFGEVFNATRFASVYAWNRKRISNCVADYSVLYNS<br>ASFSTFKCYGVSPTKLNDLCFTNVYADSFVIRGDEVRQIAPGQTGKIADYNYKLPDDFTGCVIAWNSNNLDSKVGGNY<br>NYLYRLFRKSNLKPFERDISTEIYQAGSTPCNGVEGFNCYFPLQSYGFQPTNGVGYQPYRVVVLSFELLHAPATVCGP<br>KKSTNLVKNKCVNFNFNGLTGTGVLTESNKKFLPFQQFGRDIADTTDAVRDPQTLEILDITPCSFGGVSVITPGTNTSN<br>QVAVLYQDVNCTEVPVAIHADQLTPTWRVYSTGSNVFQTRAGCLIGAEHVNNSYECDIPIGAGICASYQTQTNSPGSA<br>SSVASQSIIAYTMSLGAENSVAYSNNSIAIPTNFTISVTTEILPVSMTKTSVDCTMYICGDSTECSNLLLQYGSFCTQLNR<br>ALTGIAVEQDKNTQEVFAQVKQIYKTPPIKDFGGFNFSQILPDPSKPSKRSFIEDLLFNKVTLADAGFIKQYGDCLGDIA<br>ARDLICAQKFNGLTVLPPLLTDEMIAQYTSALLAGTITSGWTFGAGAALQIPFAMQMAYRFNGIGVTQNVLYENQKLIA<br>NQFNSAIGKIQDSLSSTASALGKLQDVVNQNAQALNTLVKQLSSNFGAISSVLNDILSRLDPPEAEVQIDRLITGRLQSL<br>QTYVTQQLIRAAEIRASANLAATKMSECVLGQSKRVDFCGKGYHLMSFPQSAPHGVVFLHVTYVPAQEKNFTTAPAIC<br>HDGKAHFPREGVFVSNGTHWFVTQRNFYEPQIITTDNTFVSGNCDVVIGIVNNTVYDPLQPELDSFKEELDKYFKNHT<br>SPDVDLGDISGINASVVNIQKEIDRLNEVAKNLNESLIDLQELGKYEQGSGYIPEAPRDGQAYVRKDGEWVLLSTFLGR<br>SGGGLVPQQSGDSATHIKFSKRDEDGKELAGATMELRDSSGKTISTWISDGQVKDFYLYPGKYTFVETAAPDGYEVA<br>TAITFTVNEQGQVTVNGKATKGDAHI

* This amino acid sequence includes a single chain Fc purification tag (see reference 38).
